# Hybrid AI Models for Short-Term Photovoltaic Forecasting: A Systematic Review of Architectures, Performance, and Deployment Challenges

**DOI:** 10.3390/s26061793

**Published:** 2026-03-12

**Authors:** Joan M. Saltos, M. Gabriela Intriago Cedeño, Ney R. Balderramo Velez, Germán T. Ramos León, A. Cano-Ortega

**Affiliations:** 1 Department of Electrical Engineering, University of Jaén, 23071 Jaen, Spain; jsg00073@red.ujaen.es (J.M.S.); mgic0001@red.ujaen.es (M.G.I.C.); nrbv0001@red.ujaen.es (N.R.B.V.); 2 Department of Electrical Engineering, University Technical of Manabí, Portoviejo 130111, Ecuador; german.ramos@utm.edu.ec

**Keywords:** forecasting, PV, short-term, hybrid models, artificial intelligence

## Abstract

The rapid incorporation of solar energy (PV) systems into electrical grids has increased the demand for accurate short-term forecasts to ensure stability and improve processes. Although hybrid artificial intelligence (AI) models are increasingly being suggested to address this challenge, there is a lack of systematic compilation of their structures, effectiveness, and readiness for use in real-world applications. This paper provides a detailed analysis of 58 peer-reviewed articles (2020–2025) focused on hybrid models for short-term (1–24 h) solar photovoltaic power forecasting. We propose an innovative classification that groups hybrids into four categories: AI-AI (28%), AI with optimization (21%), decomposition-based (17%), and image-based (7%). Our research indicates that weather conditions (34%) and historical photovoltaic energy records (32%) are the most frequent inputs, and that optimized hybrids and those using decomposition achieve the best balance between effectiveness and computational efficiency. From a geographical perspective, the study focuses mainly on the United States (29%) and China (22%), suggesting that more extensive climate validation is crucial. Essentially, we have identified ongoing obstacles to implementation, such as high computational costs, data quality issues, and gaps in interpretation. In addition, we present a plan for future research focusing on hybrid architectures that are lightweight, understandable, and interactive with the grid. This analysis provides a thorough assessment of the current landscape and a strategic framework to guide the creation of operational forecasting systems capable of supporting highly solar-integrated grids.

## 1. Introduction

**Figure 1:**
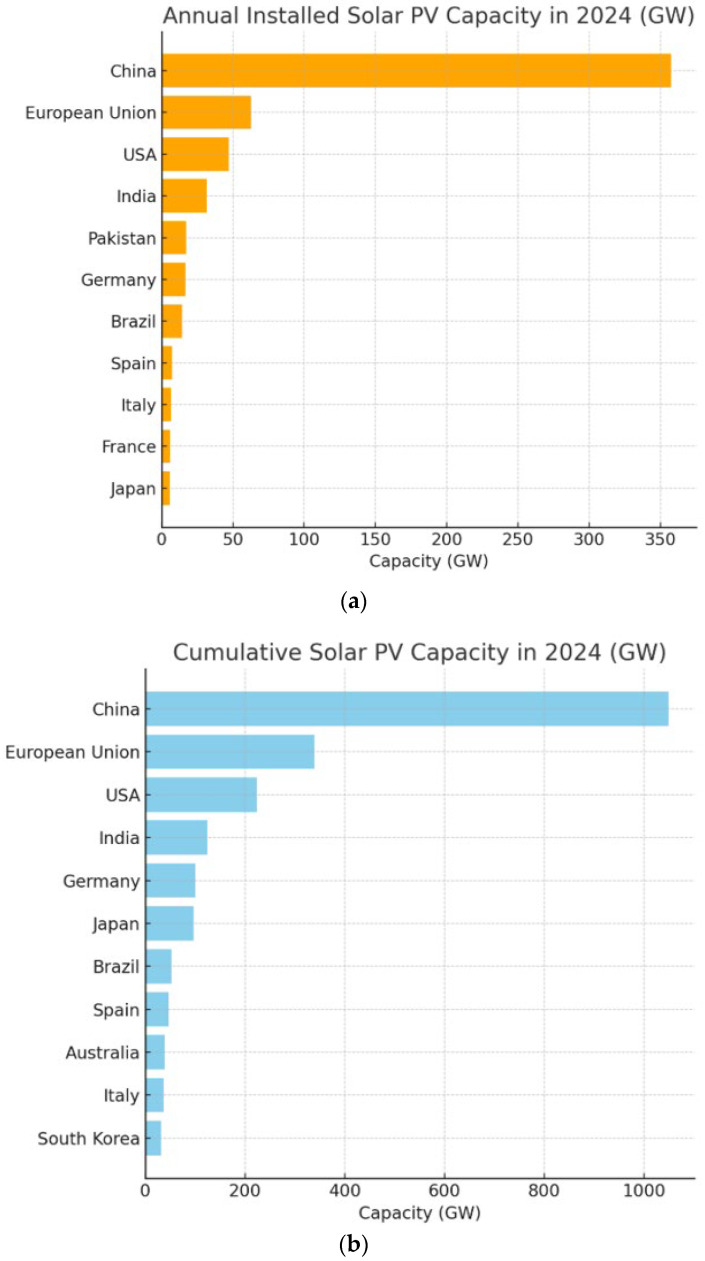
(**a**) Annual installed solar PV and (**b**) Cumulative solar PV in 2024 around the world.

Solar photovoltaic energy has emerged as one of the fastest-growing renewable electricity technologies and has great potential worldwide [1]. According to recent estimates by the International Renewable Energy Agency (IRENA), global photovoltaic solar energy capacity is expected to reach 5234 GW by 2030, indicating significant growth and accelerating the shift toward low-carbon energy systems. Figure 1a illustrates installed capacity per year in 2024, and Figure 1b illustrates total cumulative capacity for 2024 [2].

However, the natural fluctuation of solar radiation generates irregularities that impact the reliability and consistency of electrical systems, especially in situations where there is a large incorporation of renewable energies [3]. This challenge has increased the need for more accurate, robust, and flexible forecasting techniques for solar photovoltaic energy, especially for time periods in which operational decisions react significantly to variations [4,5].

In this field, hybrid models have become one of the most promising areas of study [6]. Recent research indicates significant advances in the accuracy of predictions [7], reduction in economic losses and lower costs due to penalties [8], efficient transfer of attributes in situations with limited data [8], and optimization in daily dispatch [9].

These approaches integrate information acquired through irradiance sensors, weather sensors, electrical sensors, and image-based sensors, allowing for a more comprehensive characterization of the dynamic behaviour of photovoltaic systems [10]. Several studies have demonstrated that incorporating multisensory data into hybrid architectures significantly reduces errors in multiple performance metrics [11], improves the stability of predictions under variable atmospheric conditions [12], and enhances the ability of models to adapt to real operating environments with high solar penetration.

In addition to atmospheric variables, a growing number of studies incorporate electrical sensors, such as current, voltage, and active power, as fundamental inputs in hybrid photovoltaic prediction models. These variables, measured directly in inverters, smart meters, and SCADA systems, exhibit a high correlation with the actual operating state of the PV system, enabling the capture of losses, thermal effects, and performance degradation that cannot be inferred solely from irradiance. Recent studies demonstrate that integrating historical electrical data with meteorological variables and deep learning techniques improves the accuracy of short-term forecasts and enables advanced applications such as self-consumption management, optimal dispatch, and intra-day planning [5,13]. In this context, electrical sensors act as a direct link between the physical behavior of the system and predictive models based on artificial intelligence.

Complementary to this, image-based sensors, such as sky cameras and satellite sensors, have become a key tool in modern photovoltaic prediction approaches, particularly in ultra-short-term horizons. These sensors enable the capture of the spatial and temporal evolution of cloudiness, providing anticipatory information that is not available through traditional point sensors. Recent literature highlights that hybrid models that combine image data with convolutional and recurrent neural networks achieve substantial improvements in irradiance and PV power prediction compared to approaches based exclusively on weather sensors [14,15,16]. Consequently, vision sensors constitute an essential component of new-generation hybrid architectures, reinforcing the predictive capacity of models in highly dynamic scenarios [17].

Their increased importance is attributed to the natural limitations of physical, statistical, and machine learning methods when used in isolation [18]. Hybrid structures combine the complementary benefits of numerical weather prediction, statistical learning, and artificial intelligence, facilitating better performance in different weather situations [19].

Despite their capabilities, hybrid models still face various technical and operational challenges, such as the appropriate choice of fundamental models, interference and anomalies in irradiance and photovoltaic energy measurements, uncertainty in climate predictions, unreliable confidence signals, complicated hyperparameter tuning, rapid climate change, and high computing costs [20].

An organized, quantitative, and [14,15,16] architecture-focused collection of hybrid models for short-term photovoltaic energy forecast (1–24 h) is provided in this review. The primary contributions are as follows:

The first comprehensive comparison in terms of accuracy, computational cost, complexity, and data needs is provided by classifying four major classes of hybrid models from 58 recent investigations.

A clear quantitative perspective on the evolution of methodologies is provided by the fact that 28 percent of studies combine artificial intelligence with other artificial intelligence, 21 percent combine artificial intelligence with optimization techniques, 17 percent use hybrids that break down signals, and 7 percent use image-based techniques.

Climate variables, historical solar energy production, and irradiance indices are the most important data, which explains how high-performance models are constructed, according to the grouping of 14 parameter groups.

The United States 29% and China 22% account for almost 51% of the world’s contributions, whereas Australia, Morocco, and India are significant regions for validation in various climatic conditions.

Evidence indicates that optimized and decomposition-based hybrids provide the best balance between accuracy and robustness, while image-based hybrids are superior for very short-term forecasts.

The review is organized around four research questions (RQs), each focusing on a key aspect: (1) the input parameters and hybrid architectures used in PVPF models, (2) the impact of hybrid architectures on predictive performance, (3) the limitations and challenges associated with input data, and (4) barriers to real-world implementation, along with (5) future research directions. By combining technological developments, limitations, and emerging trends, this study provides a comprehensive guide for academics and experts involved in renewable energy forecasting and grid integration tactics.

By presenting organized information on technological advances, shortcomings and future prospects, this study serves as a useful reference for researchers and experts focused on renewable energy management and its integration into smart grids. Current reviews on solar forecasting have provided important overviews of statistical, machine learning, deep learning, and numerical weather prediction techniques, as well as image-based approaches, along with their forecasting horizons and practical applications. However, they only provide a superficial and fragmented analysis of hybrid models. The present reviews fail to clearly classify hybrid architectures, compare their integration methods, or examine their advantages and disadvantages in comparison with independent methods. A methodical and focused review of hybrid forecasting strategies is still needed to structure the field, highlight performance advantages, and guide the development of more accurate and robust hybrid models for the next generation. Table 1 shows a comparative analysis of recent literature on photovoltaic forecasting. The rest of this document is organized as follows: Section 2 describes the methodology used to select and evaluate the literature; Section 3 sets out the theoretical foundations of solar energy hybrid forecasting; Section 4 examines the input parameters and hybrid architectures identified; Section 5 reviews enabling technologies and their limitations; Section 6 analyses the results in terms of performance; Section 7 presents a critical analysis of the implications and limitations; and Section 8 summarizes the conclusions and suggests future directions for advanced hybrid models suitable for real-world energy systems.

## 2. Methodology

**Figure 2:**
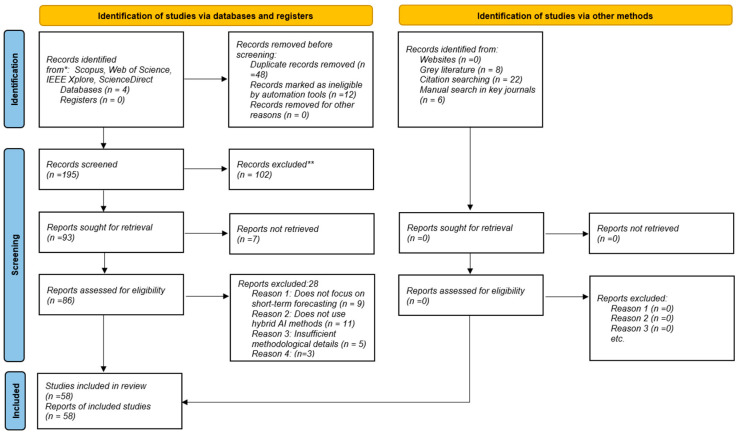
Complete screening workflow, including reasons for exclusion at each stage. * Consider, if feasible to do so, reporting the number of records identified from each database or register searched (rather than the total number across all databases/registers). ** If automation tools were used, indicate how many records were excluded by a human and how many were ex-cluded by automation tools.

This systematic review is based on the methodological guidelines of Kitchenham and Charters [29], which are widely recognized in the scientific community for their thoroughness, clarity and evidence-based approach [30], being part of these standards offers several significant advantages, such as the organized development of protocols, reduced bias in study selection, and greater clarity and repeatability of procedures [31]. The main objective of this analysis is to compile a comprehensive and exhaustive review of the relevant literature published between 2020 and 2025, focusing particularly on short-term solar energy forecasting using hybrid techniques [32]. To ensure transparency, reproducibility, and methodological rigor, the study selection process was conducted following the PRISMA 2020 guidelines. The identification, screening, eligibility assessment, and inclusion stages are summarized in Figure 2. A total of 195 records were initially screened after removing duplicates and ineligible entries. Following title and abstract evaluation, 93 reports were sought for retrieval, of which 86 were assessed for eligibility. After applying predefined inclusion and exclusion criteria primarily focusing on short-term forecasting scope, hybrid AI methodology, and sufficient methodological detail 58 studies were finally included in the review.

**Figure 3:**
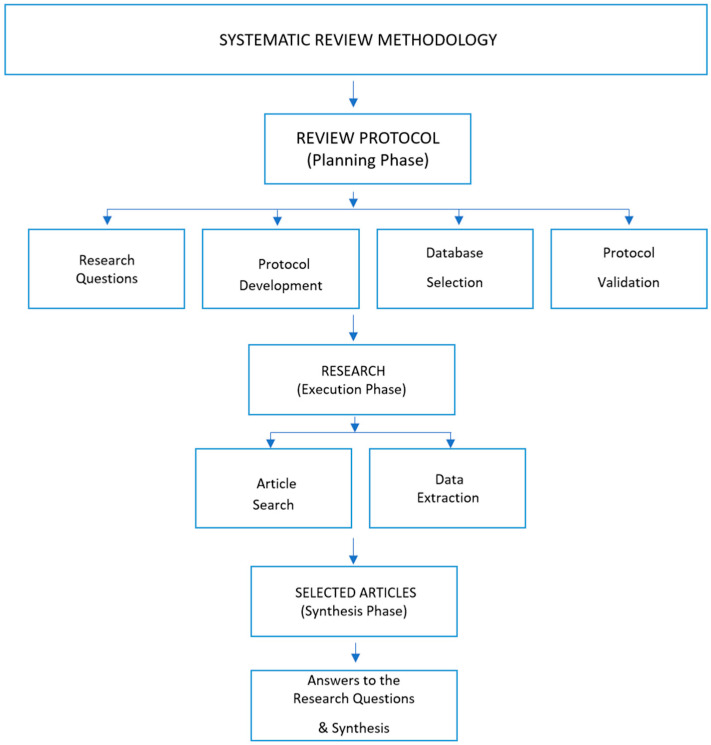
Systematic review methodology flowchart.

The methodology is organized into three main phases, as illustrated in Figure 3: (1) Development of the review protocol, (2) Literature search and execution, and (3) Selection and synthesis of studies. This structured approach ensures a transparent, replicable, and systematic process for identifying, evaluating, and synthesizing relevant research.

**Figure 4:**
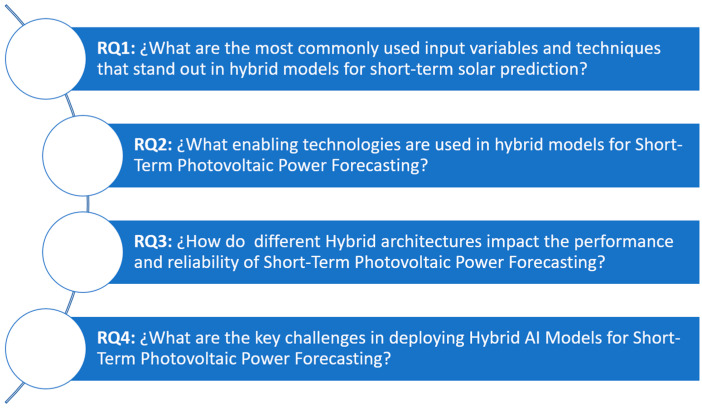
Systematic review guided by research question.

This methodological framework ensures a systematic and reproducible process for identifying, evaluating, and synthesizing relevant literature. The research questions guiding this review are illustrated in Figure 4.

A comprehensive keyword search plan was developed to systematically identify relevant literature in three main areas: (1) solar photovoltaic forecasting, (2) short-term forecasting horizons, and (3) hybrid methodologies. The search covered synonyms, acronyms, and different terminological variants in each field. To refine the search and tailor it to the study’s focus, additional terms related to modelling techniques, data processing methods, and evaluation metrics were incorporated. This method ensured the inclusion of publications relevant to the predefined research questions, while preserving methodological rigour. The final search can be found in Table 2.

Following the application of the search string to the title, abstract, and author keyword fields, a total of 661 articles were initially identified. The search was conducted using a date filter for the period 2020–2025 in ScienceDirect and IEEE Xplore. The results were limited to review articles and original research articles within the energy and engineering subject areas.

**Figure 5:**
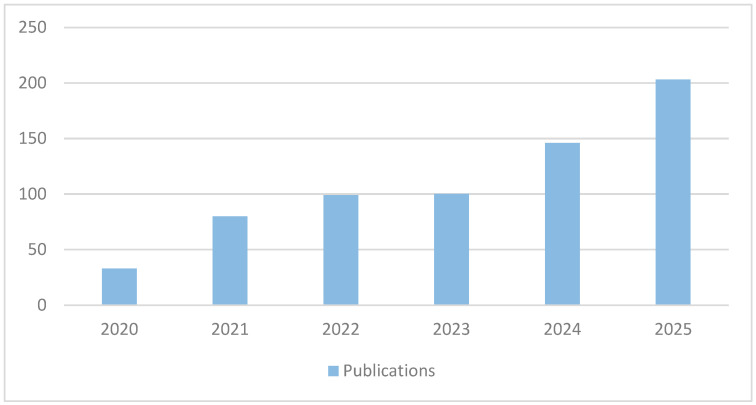
Content distribution until July 2025.

Figure 5 shows how the selected publications related to short-term photovoltaic solar energy forecasting (PVPF) using hybrid models are distributed over time and by type between 2020 and 2025. There is a clear increase in the number of publications, especially peer-reviewed articles, which are the most frequently published each year and peak in 2025. This growth reflects growing academic interest and active research in energy management using hybrid PVPF, possibly motivated by advances in artificial intelligence-driven data analysis and the growing need for sustainable energy solutions.

**Figure 6:**
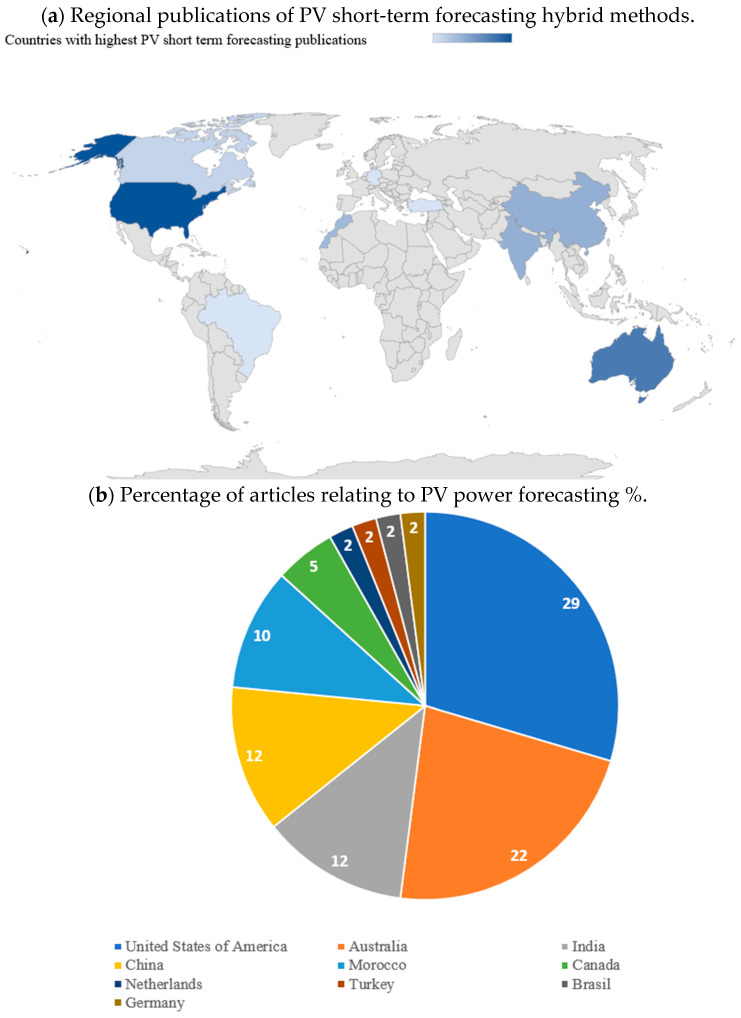
Publications pertaining to hybrid models for short-term photovoltaic energy forecasts are distributed globally and have a relative share. (**a**) Regional publications on hybrid approaches for short-term photovoltaic forecasts. (**b**) The percentage of papers by country, emphasizing the nations that make the greatest research contributions.

From a geographical perspective, only a limited number of countries are leaders in research on forecasting for hybrid photovoltaic systems. Figure 6 illustrates the global distribution of publications dealing with these models. The United States, China and India account for most of the contributions, indicating their strong commitment to incorporating renewable energies and their considerable installed capacity in photovoltaics. Australia and Morocco are also recognized as important contributors, especially due to their abundant solar resources and increased investment in solar technologies. On the other hand, countries such as Canada, Germany, the Netherlands, Turkey and Brazil show more modest, albeit relevant, efforts in advancing these prediction models.

## 3. Forecasting Models

### 3.1. Solar Forecasting

The essential characteristics of photovoltaic solar energy production are crucial for solar energy simulation and forecasting. This section explains the important aspects of solar forecasting, including the variables relevant to forecasting and the time frames for forecasting.

**(A)**  **Forecast horizon**

The forecast horizon officially describes the prospective time interval over which photovoltaic energy production is estimated. It is widely recognized in the literature that the uncertainty and magnitude of error in forecasts generally increase with the length of the time horizon [33]. Forecast horizons are conventionally classified into four main categories:

1. Very short-term forecast: Predictions ranging from seconds to minutes, and typically not exceeding 30 min [34].
2. Short-term forecast: Predictions covering periods from 1 h to 48 h, or up to a week in advance [35].
3. Medium-term forecast: Projections covering a period ranging from one week to one month in advance [36].
4. Long-term forecast: Estimates covering a period ranging from one month to one year in advance. These forecasts play a key role in strategic planning for electricity generation, transmission infrastructure development and distribution network management [37].

**Figure 7:**
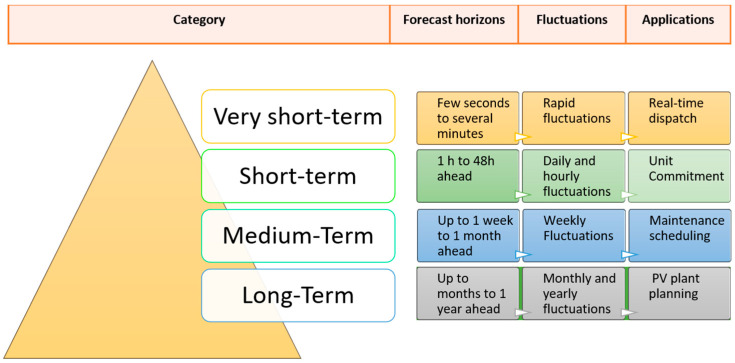
Predictive horizon and decision-making-related activities.

The corresponding applications for decision-making are shown schematically in Figure 7.

**Figure 8:**
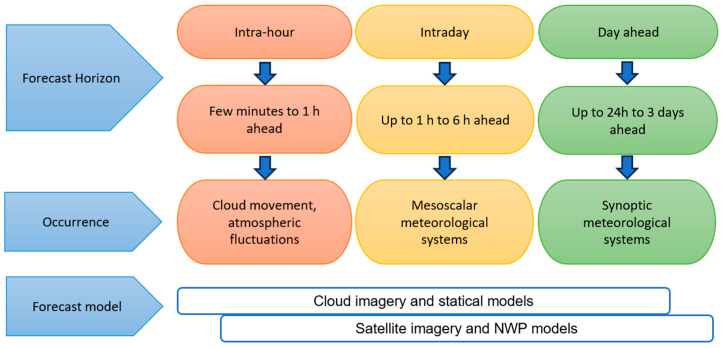
Models, activities, and outlook.

Short-term photovoltaic (PV) forecasting is defined as the estimation of energy production over a period of 60 min to 6 h [38]. This temporal division is based on the characteristics of meteorological processes that influence solar irradiance at different scales [39]. In the range of one minute to one hour (short-term forecast), the movement of individual clouds and short-term fluctuations in atmospheric turbulence dominate [40]. In the range of one to three hours (very short-term forecast), mesoscale weather systems and local convection patterns exert a significant influence. Over a period of three to six hours (short-term forecast), synoptic systems increasingly determine the behavior of solar irradiance [41]. This temporal stratification determines the most appropriate modelling techniques for each period. Methods based on persistence and satellite imagery are optimal for immediate forecasts, while numerical weather prediction (NWP) models and machine learning (ML) techniques are more effective for longer forecast periods [42]. Figure 8 illustrates the relationship between short-term forecast horizons, forecast models, and dominant solar intermittency phenomena [43].

**(B)**  **Spatial Dimension in Short-Term Photovoltaic Forecasting**

In addition to temporal aspects, consideration of space is vital to the accuracy and robustness of solar energy prediction models. The geographical scale used for prediction, whether at the level of individual solar installations or broader regional and grid aggregations, has a notable impact on patterns of variability and model effectiveness.

When focusing on individual plants, the accuracy of predictions is heavily influenced by local weather behavior, such as rapid changes in cloud cover, differences in microclimates, the influence of shading, and variations in sunlight received at specific locations. In these cases, the use of high-resolution data, such as sky cameras, ground-based solar sensors, and specific meteorological data, is crucial. Hybrid approaches at this level typically require a variety of inputs and systems that can respond quickly to accurately reflect variability within an hour [44]. Therefore, effective hybrid PV forecasting systems should explicitly consider spatial granularity as a design parameter, aligning model complexity, input features, and computational architecture with the intended deployment scale.

In comparison, forecasting on an aggregate or regional basis gains advantage from the effects of spatial smoothing. When several solar power setups are spread out over an extensive region, localized changes in sunlight often counterbalance one another, leading to a decrease in overall variability and resulting in diminished normalized forecasting inaccuracies. Consequently, more straightforward hybrid configurations can perform effectively on a regional level in comparison to highly intricate models focused on individual plants [45]. Nevertheless, forecasting on a large scale introduces fresh obstacles, including the need for modeling spatial correlations, synchronizing data among a network of distributed sensors, and coordinating with energy management systems at the transmission level. Inputs obtained from satellites and numerical weather prediction (NWP) models are becoming increasingly significant for broader geographic scopes.

Thus, efficient hybrid PV forecasting systems need to take spatial granularity into account as a design factor, ensuring that model complexity, characteristics of inputs, and computational structure are aligned with the scale of deployment intended [20].

### 3.2. Photovoltaic Solar Power Forecasting Techniques

The forecasting of solar energy production or solar irradiance is a non-linear problem influenced by meteorological conditions [46]. Identifying parameters that accurately capture these non-linear trends is challenging. The process of forecasting photovoltaic (PV) energy production generally consists of three main steps. First, energy properties are extracted and influencing factors are analysed. Next, a suitable forecasting method is selected and the model is optimized [47]. Photovoltaic forecasting methods can be classified into three groups according to the methodology used: physical, statistical, and hybrid. This section briefly describes the different solar forecasting methods, focusing on hybrid approaches.

**(1)**  **Statistical models**

**Figure 9:**
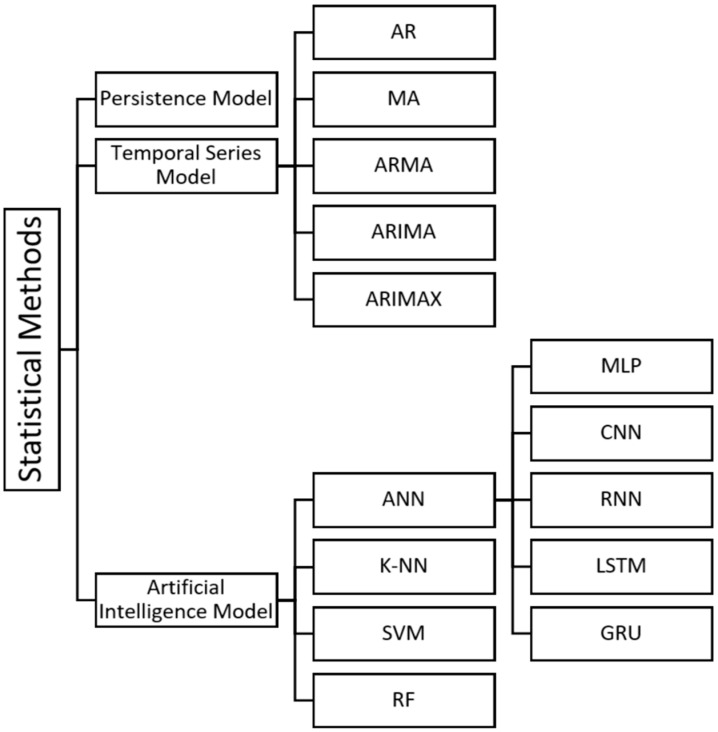
Categorization of static techniques.

Prediction techniques based on previous solar radiation records are classified into two groups: statistical techniques and learning techniques. Examples of statistical techniques include the study of seasonality, ARIMA models, multiple regression, and exponential smoothing. In contrast, artificial intelligence approaches encompass fuzzy inference systems, genetic algorithms, neural networks, and machine learning, among others. Figure 9 shows a categorization of statistical models according to their fundamental principles.

This classification summarizes the diversity of approaches used in statistical methods for short-term photovoltaic energy forecasting.

Statistical models are trained on available data to extract relevant patterns that enable accurate predictions. The quantity and quality of data are the most influential factors in forecast accuracy. The two main categories of data-driven models are time series (TS) and machine learning (ML) models [48]. AI-based statistical models are widely used for solar energy forecasting [49]. As a result, various intelligent computing technologies are increasingly employed as alternatives to conventional techniques. Table 3 compiles recent publications that use AI models for PV power forecasting, outlining their temporal horizon, temporal resolution, input parameters, forecasting models, and key insights.

Based on the information presented in Table 3, various AI models demonstrate a high capacity for capturing nonlinear relationships in solar power forecasting. However, their performance is dependent on large volumes of data and substantial computational resources.

ANN-based approaches have emerged as a prominent alternative for modelling complex nonlinear relationships between climatic variables and power generation. The following section synthesizes relevant applications and findings of ANN models used for short-term PV forecasting, with a focus on key variations including MLP, RNN, CNN, LSTM, and GRU architectures.

CNN models are widely applied in the energy sector for short- and medium-term solar radiation prediction [67]. Radio neural networks (RNNs) are frequently used to estimate solar irradiance predictions, achieving 97% accuracy with an RMSE of 4% [58]. Deep multi-layer configurations, especially LSTM networks, are used to forecast short-term energy generation [68]. Using past records of solar irradiance, solar energy, and numerical climate predictions, a GRU-based model has been created to forecast long-term solar energy [69]. For meteorological variables such as temperature and solar irradiance, the K-NN algorithm, based on pattern recognition, is a reliable method for predicting daily sequences from historical meteorological data [70]. SVM is used to estimate photovoltaic power using a time series analysis approach [71]. RF is applied for the prediction of photovoltaic energy generation [72].

**(2)**  **Physical models**

**Figure 10:**
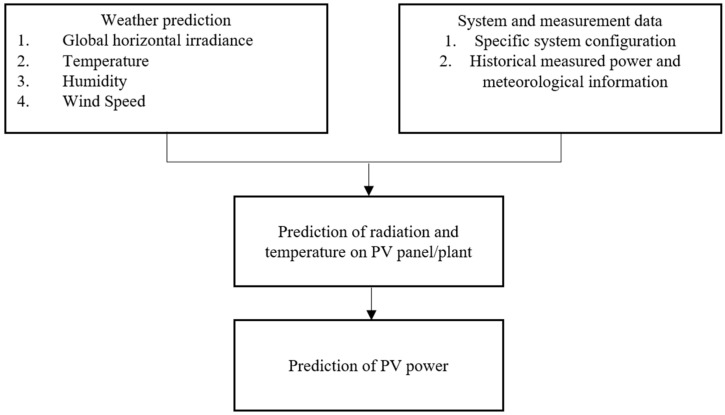
Generalized framework for physical model-based photovoltaic (PV) energy prediction.

Physical models represent how photovoltaic (PV) modules transform solar energy into electricity. These models estimate daily energy production based on anticipated weather data for a specific day [73]. The essential input elements include solar radiation, cloud cover and ambient temperature. Mathematical equations are used to determine the expected amount of energy generated by the solar panels [74]. Scientists can customize these models for different locations by incorporating local information such as panel tilt, particular weather patterns in the area, and historical energy generation data [75]. However, these physical models are most efficient under stable weather conditions; sudden fluctuations in weather variables can affect their accuracy. One example of a physical forecasting method involves the use of satellite imagery. This technique calculates solar energy production by analysing surface solar radiation records obtained by satellites in geostationary orbit [76]. Figure 10 presents the general outline of the physical models, which integrate climate forecasts with system information and measurement data to model solar irradiation on photovoltaic panels and anticipate the generated energy.

Figure 10 highlights how prediction based on physical models depends heavily on the accuracy of meteorological data and system characterization.

**(3)**  **Hybrid models**

The essential concept of hybrid methods is based on combining models with different theoretical foundations and predictive capabilities. This collaboration allows these approaches to address different aspects of solar variability that influence the accuracy of forecasts at various time scales and in different climatic situations [77]. By combining complementary techniques, hybrid models offer a more detailed description of the natural uncertainty of meteorological and photovoltaic systems, as well as providing greater adaptability to changing weather conditions. For example, scientists have created models that fuse deep neural networks with numerical weather prediction models, leveraging both the generalization capabilities of artificial intelligence and the physical basis of atmospheric modelling [78].

Hybrid artificial neural network (ANN) models that use metaheuristic approaches combine the predictive power of ANNs with the optimization capabilities of metaheuristic algorithms. This approach aims to overcome the limitations of ANNs alone, particularly their susceptibility to getting stuck in local minima during training and their sensitivity to initial parameters and network architecture [79]. Long short-term memory (LSTM) and gated recurrent unit (GRU) recurrent neural networks have demonstrated high efficiency in forecasting applications [80]. To address the challenges associated with search and convergence in inverse problems based on evolutionary computation, optimizations have been applied using hybrid models that employ techniques such as Flower Search Optimizations (FSO) and Particle Swarm Optimisation (PSO) [81]. Methods for identifying optimal deep learning model parameters using Genetic Algorithms (GAs) are also widely adopted for short-term forecasting [82].

Table 4 summarizes a selection of studies from 2020 to 2025 on short-term, very short-term, intra-hourly and daily photovoltaic energy forecasts, with an emphasis on hybrid approaches and AI-based model optimization techniques. Each entry is classified by year of publication, time horizon and resolution, input parameters, forecasting model, country of origin of the photovoltaic data, and relevant observations. This compilation facilitates the identification of methodological and regional trends in photovoltaic forecasting, allowing for the comparison of hybrid approaches in different climates and data sources, and highlights improvements in accuracy relative to reference models.

### 3.3. Domain Applications of Short-Term Photovoltaic Forecasting

Short-term forecasting of electricity generated by solar panels is proving to be a key issue for the management and design of electrical systems affected by fluctuating renewable energy sources. As the amount of decentralized solar energy grows in different geographical areas, the ability to accurately forecast electricity production over periods ranging from a few minutes to a few hours is proving crucial to maintaining grid stability, optimizing operational efficiency, and ensuring system security [23].

Intra-hourly forecasts are fundamental for frequency and voltage management, as well as for automatic generation management in electrical systems with a high integration of solar energy [127]. In today’s grid environments, these forecasts contribute directly to Automatic Generation Control (AGC) and Energy Management Systems (EMSs), enabling real-time dynamic realignment in response to variability. Furthermore, short-term expectations are used to improve the scheduling of thermal power units, battery utilization, and energy storage strategies in microgrids (MGs) and isolated systems [103].

Very short-term projections are especially necessary to ensure real-time balance between load, generation, and storage, particularly in hybrid configurations where multiple distributed energy sources operate simultaneously [128].

In expansive solar power facilities, the incorporation of hybrid AI frameworks into SCADA systems is becoming more prevalent for the purposes of ongoing surveillance, detecting anomalies, and planning operations for the following day. In such industrial settings, simply having accurate predictions is not enough; forecasting systems must also adhere to stringent latency standards, maintain high dependability, and show robustness against issues like sensor drift, measurement inaccuracies, and disruptions in communication. Furthermore, aligning varied data feeds from weather sensors, electricity meters, and satellite or aerial imaging systems poses significant technical difficulties within distributed IoT-Cloud setups.

In the context of microgrids and remote energy networks, forecast results must be closely integrated with Battery Management Systems (BMSs) and inverter control methods to ensure immediate power equilibrium while reducing dependence on fossil fuel backup generation. This integration necessitates that forecasting models are not only precise but also efficient in computation and able to function on limited-resource edge devices that are deployed on-site.

Models utilizing satellite-based deep neural networks (DNNs) that can adapt across various PV locations without needing local measurement tools have been suggested for areas with minimal infrastructure. These methods have shown prediction inaccuracies that are on par with those from models trained locally, enabling broader application in developing regions where monitoring systems are scarce. From an industrial angle, this ability to generalize lessens installation expenses, streamlines system setup, and boosts scalability.

Beyond assessing theoretical performance, the actual execution of hybrid forecasting models in working photovoltaic facilities involves tackling further engineering challenges, such as the following:

- Interoperable integration with SCADA systems and grid management platforms.
- Compliance with grid codes related to active and reactive power control.
- Curtailment management under grid congestion conditions.
- Predictive operation and maintenance (O&M) through deviation analysis between forecasted and actual generation.
- Secure and reliable data transmission within distributed IoT architectures.

Thus, the practical use of hybrid AI systems for short-term solar forecasting in industry requires a holistic methodology that melds algorithmic precision, computational adeptness, compatibility, durability, and scalability. These factors elevate short-term forecasting beyond mere statistical modeling, making it a vital functional element in contemporary digitalized energy systems.

## 4. Internet of Things and Cloud-Based Architecture for Hybrid Solar Prediction Systems

Lately, the combination of IoT technology with photovoltaic systems has revolutionized how solar energy is observed, managed, and enhanced, allowing for instant tracking, remote operation, proactive upkeep, and optimization based on data. Essential aspects of a solar monitoring system consist of tracked variables, kinds of sensors, management units, methods of data transmission, software applications, and ways to monitor.

**Figure 11:**
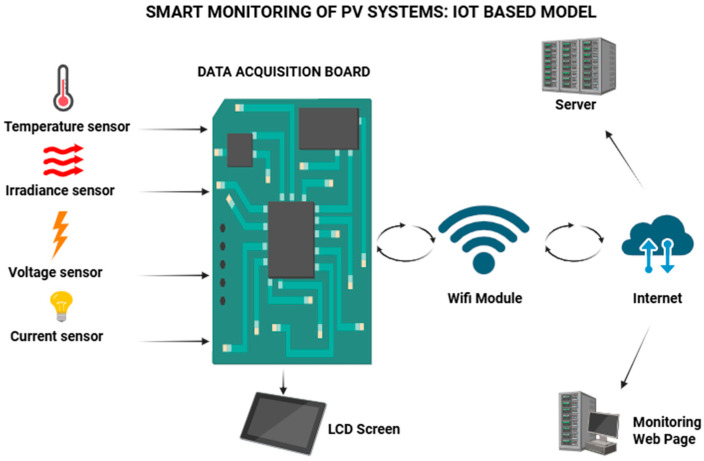
IoT-based PV monitoring system.

IoT architectures applied to energy monitoring follow a multi-layered model composed of a sensing layer, edge processing layer, communication layer, and application layer [127]. Figure 11 illustrates the block diagram of an IoT-based PV monitoring system.

### 4.1. Sensing Layer

The sensing layer consists of gathering unprocessed readings from devices that track factors like photovoltaic panel voltage, array amperage, instantaneous power, solar irradiance, module temperature, humidity levels, etc.

Hall effect sensors (ACS712), voltage dividers, LM35, and DHT sensors were connected to Arduino or ESP8266 microcontrollers for digital conversion and initial processing [127].

The integrity of the information obtained in this stage is crucial for the following training of hybrid predictive models, as continuous errors can influence the model’s ability to generalize effectively.

### 4.2. Edge Processing Layer

**Figure 12:**
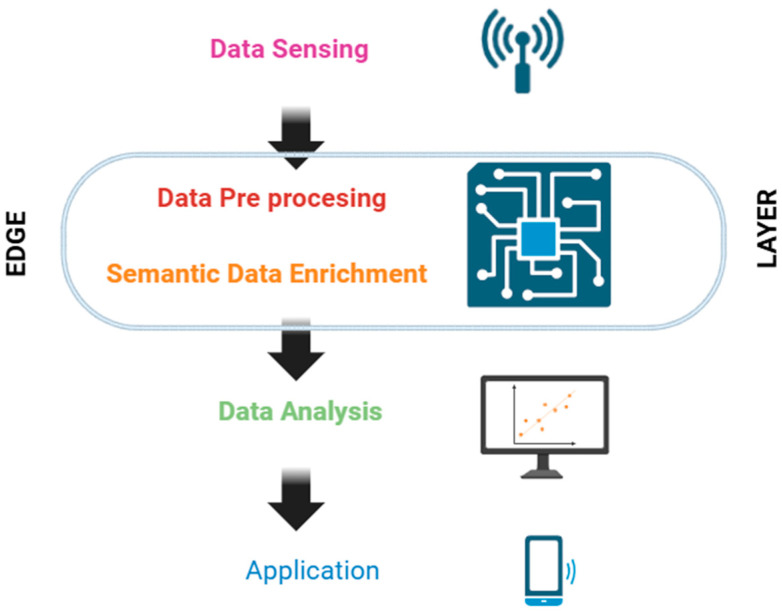
Edge Layer overview.

The system’s primary distributed architecture element is the Edge layer as show Figure 12. The PV system sensors provide the data, which is then passed to the data sensing layer for semantic enrichment and pre-processing. There is a hierarchy between the two processing sub-layers that make up the Edge layer. Since it makes sense to enrich only the qualified or properly selected data, pre-processing always comes before data enrichment. The streaming processes are then further analysed by looking more closely at each layer’s capabilities [128]. This layer can be applied on a first stage of noise reduction before feeding the hybrid physical-intelligent model.

### 4.3. Communication Layer

The communication layer of the IoT, also referred to as the network or transport layer, serves as the fundamental framework that facilitates the exchange of information among devices, gateways, and cloud services.

It accommodates a variety of protocols including Wi-Fi, BLE, Zigbee, LoRaWAN, and cellular networks like 5G, which link sensors to processing units. Essential functions of this layer encompass addressing, routing, and ensuring dependable packet delivery [127].

The selection of protocol affects the reliability of data transmission needed for immediate forecasts. Communication techniques may differ based on geographic and energy circumstances. As mentioned by [129], GSM is a more effective option for remote rural microgrids where Wi-Fi access is limited. ZigBee is employed for energy-efficient local networks as noted by [130]. In urban settings and IoT smart grid scenarios, Wi-Fi (ESP8266) is utilized. LoRa is preferred for extensive areas requiring minimal power usage.

### 4.4. Application Layer

The application layer represents the highest level within the IoT protocol framework, engaging directly with user software, applications, and devices to facilitate data formatting, assurance of security, and communication of messages [131].

The growing Internet of Things offers a chance to drastically improve the oversight of solar energy production and facility functions. For this purpose, a remote monitoring system is essential, leveraging the Internet of Things to collect and send data. This system consists of elements like a data gateway, data gathering, and display for a cloud service. Data acquired are saved in the cloud, allowing for a visual depiction of the monitored variables. To improve the use of solar energy, IoT-driven monitoring systems have been created to enable real-time data collection and analysis of solar metrics for efficiency forecasting and reliable power generation [132].

These findings highlight the necessity of considering recent climate variations when estimating solar output in near real-time, especially in isolated locations and areas that differ from worldwide patterns.

Table 5 indicates that while many IoT-based PV monitoring systems have been introduced, the majority of their designs concentrate mainly on data collection and visualization. There are few studies that incorporate edge-level processing alongside cloud-based hybrid AI forecasting models. Additionally, only a limited number of contributions assess communication reliability and its effect on the accuracy of short-term predictions. This deficiency underscores the necessity for fully integrated IoT-Edge-Cloud intelligent frameworks for photovoltaic forecasting, which serves as the primary motivation for this review.

### 4.5. System-Level Integration Challenges in IoT-Based PV Forecasting Architectures

Although IoT applications significantly improve monitoring and prediction in photovoltaic systems, there are still several system-level integration issues that have not been adequately addressed in research.

#### 4.5.1. Communication Latency and Forecasting Accuracy

Short-term photovoltaic forecasting, especially at intra-hourly and minute intervals, requires reliable real-time data flows. However, communication latency caused by wireless protocols such as Wi-Fi, GSM, and LoRaWAN can cause desynchronisation between meteorological and electrical data. Even minimal delays can negatively affect model performance, especially in hybrid artificial intelligence architectures that rely on coordinated data from multiple sources. Thus, the accuracy of predictions is not only based on the algorithm, but is also influenced by the robustness and predictability of the IoT communication infrastructure used [133].

#### 4.5.2. Packet Loss and Data Integrity

In distributed solar power systems located in rural or remote areas, problems such as packet loss and unstable connectivity are common. The lack of or imperfection of sensor data can introduce biases during the training phases and limit the ability of machine learning models to generalize.

Therefore, rigorous data validation, redundancy mechanisms, and edge-level buffering strategies are essential to ensure the quality of datasets before transferring them to the cloud [134].

#### 4.5.3. Interoperability and Data Standardization

Photovoltaic monitoring systems that use IoT technology typically combine different sensors, microcontrollers, connection devices, and cloud services. The absence of uniform standards in data formats and communication interfaces complicates communication between dispersed nodes.

To achieve seamless integration, it is essential to use standardised protocols (such as MQTT and HTTP/REST) and organised formats (such as JSON or CSV). Without adequate interoperability, the massive expansion of distributed prediction structures remains restricted [135].

#### 4.5.4. Scalability of Distributed Sensor Networks

With the rise in monitored solar power setups, the importance of system scalability grows. Centralized cloud systems might face limits in bandwidth and higher processing demands.

Hybrid edge-cloud models address this challenge by shifting initial filtering and feature extraction activities to edge nodes, lessening reliance on the cloud and enhancing the overall efficiency of the system [136].

#### 4.5.5. Cybersecurity and Data Privacy

IoT-enabled photovoltaic system structures face risks of cyberattacks, such as identity theft, data manipulation, and denial-of-service incidents. Since predictive models rely on accurate sensor data, manipulated information can lead to erroneous operational decisions.

Consequently, robust encryption, validation methods, and secure communication channels are essential elements in predictive energy systems that leverage the IoT [137].

#### 4.5.6. FAIR Data Compliance and Data Exchange

Ensuring that solar datasets produced by the IoT adhere to FAIR Standards (Findable, Accessible, Interoperable, and Reusable) is essential for reproducibility in science and collaboration in large-scale predictive research [138].

- Structured metadata annotation.
- Persistent identifiers (DOIs) for datasets.
- Open and machine-readable formats.
- Public or controlled-access repositories.

The methodical incorporation of data sources that align with FAIR Standards promotes clarity, enables better comparison of hybrid artificial intelligence models, and optimizes data exchange between different institutions.

## 5. RQ1: What Are the Input Variables and Most Commonly Used Techniques That Stand out in Hybrid Models for Short-Term Solar Forecasting?

First, as common input elements, meteorological characteristics like temperature, relative humidity, and wind speed clearly dominate. Furthermore, historical photovoltaic energy production data are frequently utilized.

**Figure 13:**
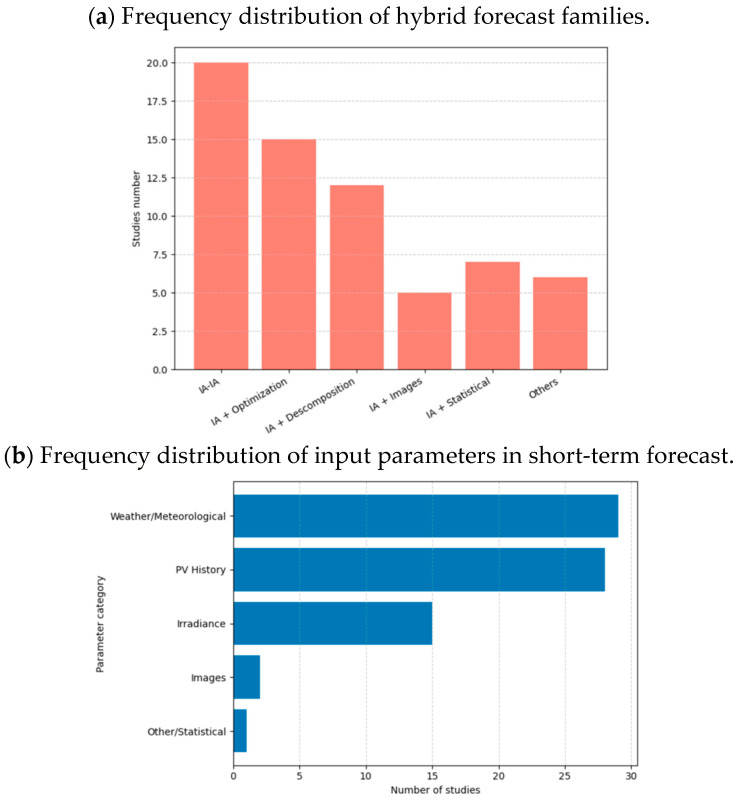
Short-term solar energy forecasting: distribution of input parameters and hybrid forecast model types. The distribution of input parameter categories and the frequency of hybrid model families.

It should be mentioned that the most popular hybrid models are AI-AI combinations, which are followed by AI combinations with signal decomposition and optimization algorithms. The distribution of the hybrid model categories and the relative frequency of the input factors utilized in the study are compared in Figure 13.

Figure 13b shows solar irradiance as a third important factor, while images and other statistical data are used less frequently as input parameters in hybrid models. This highlights the four most commonly used parameters in the development of hybrid models.

**Figure 14:**
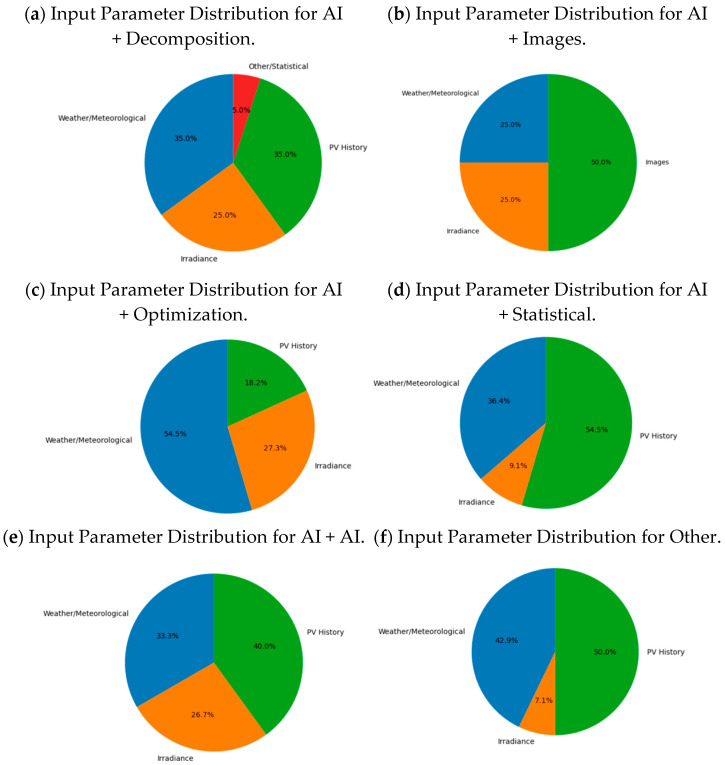
Categories of input parameters are distributed among hybrid model families for short-term photovoltaic forecasts. (**a**) Distribution of IA input parameters and decomposition. (**b**) Distribution of IA input parameters and images. (**c**) Distribution of IA input parameters and optimization. (**d**) Distribution of IA input parameters and statics. (**e**) Distribution of IA and IA input parameters. (**f**) Distribution of input parameters for other kinds of hybrid models.

By gathering information on the input parameters and the different hybrid modelling techniques used, Figure 14 shows the distribution of input parameters across different categories of hybrid models. This perspective offers a deeper understanding of hybrid modelling strategies, revealing that the choice of input data influences the effectiveness and applicability of each hybrid forecasting approach.

## 6. RQ2: What Enabling Technologies Are Used in Hybrid Models for Short-Term Photovoltaic Energy Prediction, and What Are Their Limitations?

The most widely used technologies, according to the information obtained from the analysis, reveal that deep learning architectures form the computational core of most hybrid models, and their effectiveness is often improved through evolutionary optimization for parameter tuning and signal decomposition for data refinement. Notably, the choice of enabling technology exhibits strong horizon-dependence: Vision-Based methods are particularly effective for minute-ahead forecasts, whereas Hybrid Statistical-ML frameworks show promise for probabilistic day-ahead predictions. Table 6 systematically categorizes the enabling technologies employed in hybrid models for short-term PV power forecasting, synthesizing their roles and limitations.

## 7. RQ3: Impact of Hybrid Architectures on the Performance of Short-Term Photovoltaic Energy Forecasting

**Figure 15:**
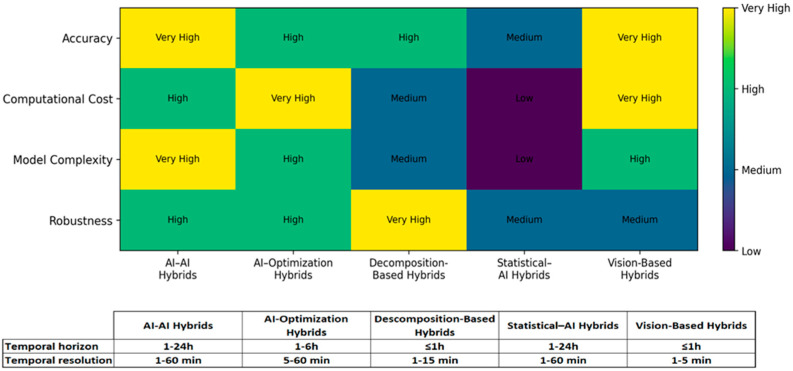
Performance metrics of hybrid model categories.

Independent models perform less well in forecasting than hybrid models. In this area, combinations of artificial intelligence with artificial intelligence predominate, especially different versions of recurrent neural networks (LSTM, GRU) that are integrated with optimization methods (GA, PSO, WOA) or signal decomposition techniques (CEEMDAN, VMD). These hybrid approaches consistently outperform individual models, with improvements in accuracy ranging from 9% to 96% in the reduction of mean square error. However, performance improvements come with inherent disadvantages. While optimization-enhanced hybrids (e.g., GWO-MLP [64]) improve accuracy by 15–30%, they increase computational cost by 40–60% due to population-based searching. Similarly, decomposition-based hybrids (e.g., EMD-SCA-ELM [70]) excel in very short-term horizons (≤15 min) but add pre-processing overhead that may not be justifiable for longer forecasts; Figure 15 summarizes the comparative performance of the main categories of hybrid PV forecasting models using a qualitative heatmap. It contrasts five model families (AI–AI, AI–Optimization, Decomposition-Based, Statistical–AI, and Vision-Based) across four dimensions: accuracy, computational cost, model complexity, and robustness. In addition, an embedded table reports the forecasting horizon and temporal resolution; since these are expressed as ranges rather than ordinal variables, they are presented separately and are not included in the heatmap.

The effectiveness of hybrid architectures is strongly horizon dependent:

- Ultra-short-term (≤1 h): Decomposition-based and vision-based hybrids perform best.
- Short-term (1–6 h): Optimization-enhanced RNNs are most effective.
- Day-ahead: Data-fusion hybrids combining NWP with AI show superior reliability.

As illustrated in Figure 15, no single architecture dominates across all metrics. AI-AI hybrids achieve peak accuracy but with high complexity, while optimization-based hybrids offer better computational efficiency, and statistical-AI hybrids provide superior interpretability.

## 8. RQ4: Challenges of Hybrid Models for Short-Term Photovoltaic Energy Prediction

The main barriers identified in the reviewed literature are detailed below, along with proposals for overcoming these observed limitations. Hybrid models combined with optimization and signal decomposition use high computational resources for training, which hinders their integration and validation in real time.

Analyses show that improved hybrids (e.g., GWO-MLP) can increase computational load by 40% to 60% compared to individual models. Furthermore, decomposition-based pre-processing introduces significant latency, particularly for very short-term forecasts. This limits scalability for utilities with limited IT budgets or facing edge deployment scenarios, especially in distributed solar energy systems.

The accuracy of predictions is based on high-quality, well-defined information (e.g., irradiance, sky photographs, PNT). Low-quality data limit the model’s ability to generalise and increases the difficulty of pre-processing, particularly in mixed models that use images and satellites.

The lack of uniformity in evaluation measures (such as RMSE, MAE, and skill score) and the variety of experimental conditions complicate fair comparison and replicability across studies. Only 34% of the studies reviewed used multiple error metrics, and less than 20% provided comprehensive quantification of uncertainty or prediction intervals.

Numerous hybrid models are created independently and are not prepared to communicate with existing SCADA, EMS, or data file systems. Only 12% of the analyses examined referenced connection to network management platforms or real-time data streams.

The following table summarizes the main barriers hindering the implementation of hybrid photovoltaic forecasting models, along with proposed solutions and supporting evidence drawn from the reviewed literature. This synthesis provides a clear roadmap for researchers and practitioners seeking to develop next-generation, grid-ready forecasting systems as show Table 7.

## 9. Conclusions and Future Directions

The literature review conducted in this paper establishes a path for discovering how the use of hybrid architectures affects short-term solar energy prediction, which is an important field for sustainable energy management companies, researchers in the field of renewable energy, and further advances in the development of energy management techniques in smart grids. Guided by four research questions, this article provides a structured and comprehensive understanding of how hybrid models are being applied, the technologies that predominate in the architectures, the challenges and limitations they face, and where the future of these models is headed.

The study begins with RQ1, which examines which of the hybrid models is the most widely used and under which input parameters learning can be performed. The analysis shows that AI-AI hybrids are the most widely used and that the input parameters for configuring these architectures are historical data and meteorological parameters. Combining AI-AI-optimization architectures can intelligently improve predictions using the same amount of input data and emerges as a promising direction.

RQ2 identifies that deep learning models are highly complex and require large amounts of data, while optimization algorithms generate high computational costs. To overcome these difficulties, we suggest creating lighter deep learning architectures for use on local devices, developing decomposition methods that adapt to specific weather patterns, and establishing benchmark standards to ensure reproducible optimization results. Furthermore, future studies should investigate federated learning models that utilize distributed solar data while maintaining privacy, as well as physics-based artificial intelligence to improve the generalization ability of models in extreme weather situations.

RQ3 addresses the impact of the family of hybrid models found in the review with regard to their performance in short-term solar energy prediction. The review highlights that AI hybrid models optimized for 1 to 6 h forecasts in advance have the highest accuracy compared to the other hybrid models found in the review. However, the computational cost is high. AI-AI hybrid models stand out for their high complexity as a model to be developed, but they are also among the models with the best accuracy without such high computational costs. Future research should develop hybrid models that combine the AI-AI family with optimization models to improve model stability while maintaining computational cost and high accuracy.

Finally, RQ4 allowed us to identify the trend towards which solutions to the complexities presented by hybrid models should be directed. The review proposes a hybrid model architecture that combines CNN-LSTM optimized with GWO, which includes the optimization of historical data processing in order to improve the model’s learning trends. This architecture aligns with the growing need for more accurate prediction systems that can not only process historical data but also use images to strengthen predictions with more input parameters.

To conclude the contributions presented in this review, a research roadmap based on the four research questions is included. Current applications of hybrid models focus mainly on improving the accuracy of their forecasts, taking into account that the complexity of the model increases and so does the computational cost. Signal processing technologies or the use of optimised models are proposed as a solution. Based on these findings, future research should aim to (i) expand the applications of hybrid models for use in the medium and long term, (ii) publish real cases through research using the hybrid models found and focus on practical applications to bridge the gap between academic research and industrial practice, (iii) develop CNN-LSTM-GWO hybrid model architectures that are adaptive to different dynamic climatic environments, (iv) promote models that standardize data collection and the evaluation of different existing architectures. This guide seeks to orient future initiatives towards hybrid models that are more scalable, intelligent, and accurate and less complex.

## Figures and Tables

**Table 1:** Comparative analysis of recent review literature on Hybrid AI Models for PV forecasting.

Refs	Year	Focus Area	Forecast Horizon	Hybrid Models	Input Features	Accuracy Metrics	Limitations
[21]	2016	Solar power forecasting: advancements & trends	Multiple horizons (seconds to days)	×	✓	✓	Comprehensive but dated; focuses on statistical and parametric methods, with limited coverage of AI, hybrid models, and modern deep learning techniques.
[22]	2017	Solar irradiation forecasting using ML	Multiple horizons	-	✓	✓	Reviews traditional ML methods (ANN, SVM, etc.) but lacks coverage of deep learning, hybrid architectures, and modern AI–AI or optimization hybrids.
[23]	2021	Intra-hour solar forecasting	Intra-hour (minutes to hours)	✓	✓	✓	Focuses on intra-hour horizon only; limited coverage of intra-day (1–24 h) hybrid models; does not address geographical or computational trends.
[24]	2023	ML, NWP, satellite, sky imaging, hybrid models	Very short to medium-term (0 h to days)	✓	✓	-	Strong focus on economic impact; limited architectural/technical analysis; does not classify hybrid families.
[25]	2024	Advancements in solar forecasting techniques	1 min to 24 h	-	✓	✓	Focuses on AI techniques and standardization; limited systematic analysis of hybrid AI architectures (AI–AI, AI–optimization, etc.).
[26]	2024	ML, DL, NWP, satellite, sky-imager, hybrid models	Ultra-short- to medium-term (0–48 h)	✓	✓	✓	Discusses hybrid approaches broadly but lacks classification, comparative framework, and quantitative comparison.
[27,28]	2025	PV modelling techniques: ML, irradiance models, NWP	Minutes to weeks	-	✓	-	Broad narrative review; lacks structure, quantification, and identification of gaps in hybrid model research.
[29]	2025	Classical ML algorithms	Short-term (<24 h)	×	✓	✓	Limited scope (only ML); no taxonomy of hybrid approaches; lacks integration of NWP or DL perspectives.
[30]	2025	Computer vision-based forecasting	Ultra-short-term (seconds to ~30 min)	-	✓	-	Focus limited to image-based ultra-short-term forecasting; not generalizable to horizons >30 min; high hardware dependency.
This Review	2026	Hybrid AI models for short-term PV forecasting	1–24 h (Intra-day)	✓	✓	✓	Comprehensive coverage of hybrid model types (AI–AI, AI–optimization, decomposition-based, image-driven), input features, geographical trends, accuracy, robustness, and computational efficiency, with identified challenges and future directions.

× Not discussed; - Partially discussed; ✓ Well discussed.

**Table 2:** Systematic search string.

Category	Search Terms	Publications	Percentage of Total Base (%)
**Solar prediction**	(“solar power prediction” OR “photovoltaic forecasting” OR “PV prediction” OR “solar energy forecasting” OR “solar generation prediction” OR “solar irradiance forecasting” OR “photovoltaic power forecasting”)	293	44%
**Temporal horizon**	(“short-term” OR “short term” OR “intraday” OR “hourly” OR “daily” OR “ultra-short-term” OR “ultra short term” OR “very short-term”)	157	24%
**Hybrid methods**	(“hybrid method” OR “hybrid model” OR “hybrid approach” OR “hybrid technique” OR “ensemble method” OR “combined model” OR “multi-model” OR “fusion model” OR “integrated approach”)	86	13%
**Techniques**	(“machine learning” OR “deep learning” OR “artificial intelligence” OR “neural network” OR “statistical model” OR “time series” OR “regression” OR “optimization”)	125	19%

**Table 3:** Comparative analysis of AI-based forecasting approaches for short-term PV power prediction.

Ref.	Year	Temporal Horizon	Temporal Resolution	Input Parameters	Prediction Model	Key Insights
[50]	2024	Short-term	5–60 min	PV output & battery state-of-charge	Artificial Neural Networks (ANNs)	Applied to solar charging stations for EVs; effective for supply-demand balancing and advanced energy management.
[51]	2010	Short-term	24 h	Solar irradiance	Multilayer Perceptron (MLP)	Solar irradiance forecasting; improves control algorithms for grid-connected PV plants.
[52]	2014	Short-term	Daily	NOAA weather forecasts + irradiance	ANN with spatial modelling	Continuous learning of GHI profiles; useful for sites lacking local historical data.
[53]	2015	Short-term	Hourly	Solar irradiance	ANN with wavelets	Wavelet integration enhances irradiance forecast accuracy in Shanghai.
[54]	2013	Short-term	Hourly/Daily	PV power time series	ANN vs. time series models	ANN outperformed time-series methods in PV generation forecasting.
[55]	2013	Short-term	Hourly/Daily	Solar radiation under varying sky conditions	Fuzzy networks + ANN	Sky conditions did not significantly impact prediction accuracy.
[56]	2013	Short-term	Hourly	Monocrystalline panel output (50 Wp)	ANN	Superior accuracy compared to polynomial and linear regression models.
[57]	2014	Short-term	1 h	Global solar irradiance & air temperature	Dynamic Neural Networks	Effective for 1 h ahead solar power predictions.
[58]	2015	Short-term	5–30 min	Sky images	Optimized k-NN	Forecasts global (GHI) and direct (DNI) irradiance with uncertainty intervals.
[59]	2021	Short-term	1 h	Solar irradiance, meteorological data	k-NN, LR, ANN, SVM	Comparison of supervised algorithms for PV power forecasting in Medellín, Colombia.
[60]	2012	Short-term	Hourly	Historical solar generation data	k-NN, ARIMA, ANN	Evaluation of forecasting techniques without exogenous inputs for a PV plant in Merced, California.
[61]	2014	Short-term	1 day ahead	Meteorological data	SVM	Comparison of methods for day-ahead PV power forecasting.
[62]	2015	Short-term	Hourly	Solar irradiance, meteorological data	SVM	Pattern recognition-based SVM model for short-term PV power forecasting.
[63]	2019	Medium-term	1–6 days	Solar radiation	Random Forest (RF)	Comparison of models for solar radiation prediction in Gorakhpur, India.
[64]	2024	Short-term	1 day	Operational data from a PV system	Individual & hybrid AI models	Evaluation of models for PV output forecasting using real operational data.
[65]	2024	Short-term	Hourly	Electrical load data	RF + density-based clustering	Load forecasting model considering noise and consumption patterns.
[66]	2015	Short-term	Hourly	Solar irradiance, meteorological data	SOM + SVR + PSO	Hybrid approach using Self-Organizing Maps for solar irradiance forecasting.

**Table 4:** Hybrid and optimized AI models applied to short-term photovoltaic forecasting: A comparative compilation (2020–2025).

Ref.	Year	Temporal Horizon	Temporal Resolution	Input Parameters	Prediction Model	Country	Key Insights
[83]	2020	Short-term	6 h ahead	Meteorological data	Optimized ANN	Morocco	Accurately forecasts global horizontal irradiance (GHI). The proposed HAEANN models outperformed SP, RT, and RF models in all tested scenarios.
[84]	2020	Ultra-short-term	15 min ahead	Historical solar energy data	CSO-RBF	Netherlands	Applied to solar energy forecasting. The optimized competitive radial basis function neural network achieved higher accuracy compared to similar models.
[85]	2023	Short-term	1 h ahead	Meteorological data, shortwave radiation (SRAD 1)	ADCMA	USA	Solar radiation forecasting. An effective evolutionary hyperparameter tuner (ADCMA) enhances solar radiation prediction accuracy.
[86]	2020	Short-term	1 day ahead	Meteorological data	GWO-MLP	India	Model performance for PV output forecasting surpasses other intelligent techniques. Useful for demand response in smart grid environments.
[87]	2020	Short-term	1 day ahead	Air temperature, relative humidity, total & diffuse horizontal solar radiation	GWO-MLP, ALO-MLP, WOA-MLP	Turkey	The GWO-based MLP model was most successful and competitive for daily PV energy prediction.
[88]	2022	Short-term	Not specified	Meteorological data (temperature, dew point, humidity, etc.)	GWO-MLP, GWO-CNN, GWO-RNN, DHOA-MLP, DHOA-CNN, DHOA-RNN,8GU-DHOA-MLP, GU-DHOA-CNN, GU-DHOA-RNN	USA	Hidden neuron counts in MLP, CNN, and RNN were optimized using a hybrid GU-DHOA algorithm. The proposed algorithms reliably and efficiently predict solar irradiance.
[13]	2020	Short-term	1 h ahead	Historical global horizontal irradiance (GHI) data	LSTM-GA, GRU-GA, RNN-GA	Morocco	Combining deep learning methods with genetic algorithms improves short-term solar irradiation forecasting performance and accuracy.
[89]	2022	Ultra-short-term	5, 10, and 15 min ahead	Meteorological data	LSTM-GA	USA	Forecast performance remained below 20% across all scales, effectively improving accuracy compared to persistence and benchmark methods.
[90]	2020	Short-term	24 h ahead	Cloud cover, relative humidity, temperature	ELM-PI, GBM-PI, NN-PI	USA	GBM showed better performance, though ELM advanced due to fast, efficient learning and good generalization.
[91]	2020	Ultra-short-term & short-term	1, 5, and 50 min ahead	Historical PV energy, solar radiation, cloud cover, air temperature	AR-KF, ARMA-KF, ARIMA-KF	USA	High-resolution solar radiation and PV output forecasting for summer conditions.
[92]	2020	Ultra-short-term & short-term	15, 30, and 60 min ahead	Irradiance, temperature, PV power	EMD-SCA-ELM	India	The developed EMD-SCA-ELM approach, especially the 15 min interval method, improves ELM performance in short-term PV energy forecasting.
[93]	2020	Short-term	3, 6, and 24 h ahead	Meteorological data, DNI, DHI, GHI	Optimized LSTM	India	High accuracy in solar radiation prediction indicates efficiency for optimized solar energy system design, especially in arid zones.
[94]	2020	Short-, medium-, and long-term	1, 15, 30, and 60 days ahead	Meteorological data, wavelet-based statistical features	WT-LSTM	USA	The proposed WT-LSTM-dropout model increases short-term PV energy forecast accuracy and meets real-time application needs.
[95]	2020	Short-term	1 h ahead	Hourly irradiance data	CEEMDAN–CNN–LSTM	USA	CEEMDAN decomposes complex irradiance series into simpler IMFs, enabling accurate forecasting.
[96]	2020	Short-term & ultra-short-term	5 min & 1 h ahead	PV power time series	WPD-LSTM	Australia	WPD-LSTM outperforms LSTM, GRU, RNN, and MLP across seasons and weather conditions.
[97]	2020	Short-term	1 h ahead	Meteorological & historical solar irradiance data	CNN-LSTM	USA	Effective for short-term GHI prediction, especially under cloudy and clear skies. Requires large datasets and extended training time.
[98]	2020	Very short-term	15 min ahead	Real-time PV plant meteorological data	RF-CEEMD-DIFPSO-BPNN	Canada	Hybrid model validated on a PV plant; RF-CEEMD-DIFPSO-BPNN shows promise for PV generation forecasting.
[99]	2020	Short-term	1 day ahead	DNI and temperature	LSTM-RNN-PDPP	USA	The proposed TCM-modified forecasting method is more accurate than standalone LSTM-RNN.
[100]	2020	Short-term	1 day ahead	Historical PV data	IVMD-ARIMA-IDBN	Australia	Provides a more accurate and stable model for short-term PV output forecasting, improving accuracy by 9–96% over other models.
[101]	2021	Short-term	1 day ahead	Historical PV generation data	GRUP	China	Forecasts using the proposed method achieve higher accuracy compared to NWP models.
[102]	2021	Short- & medium-term	24 h ahead	Historical PV generation data	Physical Model-VARX + D-vine Copulas	Germany	The hybrid model outperforms standalone physical or VARX models; D-vine Copulas improve statistical interdependence in forecast errors.
[103]	2021	Short- & medium-term	1, 2, 3, 5, and 7 days ahead	Daily PV production, meteorological data, daily electricity consumption	CNN-LSTM, ConvLSTM	Morocco	The proposed models utilize multivariate dataset features to improve predictions.
[104]	2021	Short-term	1 h ahead	Irradiance, meteorological parameters	WPD-CNN-LSTM-MLP	USA	The developed hybrid model outperforms others; multivariate input structure, especially future meteorological parameters, enhances accuracy.
[105]	2021	Short-term	1 h ahead	Historical solar radiation, temperature, humidity, active power	Optimized CNN	Australia	The proposed deep forecasting method delivers more accurate and reliable predictions across test points under varying weather and seasons.
[106]	2021	Very short- & short-term	1, 5, 30, and 60 min ahead	PV energy production database	CNN1D-LSTM, CNN1D-GRU	Italy	
[107]	2021	Ultra-short-term	15 min ahead	GHI time series, meteorological data, sky images	CNN–MLP	Morocco	The proposed model is suitable for short-term GHI forecasting, even under cloudy conditions.
[108]	2022	Short-term	1 day ahead	Historical PV energy data	DCGAN-QRLSTM	China	Proposed PV power forecasting performance is 17.98–18.16% higher than GPR and standalone QRLSTM models.
[109]	2022	Short-term	2 and 6 h ahead	Historical energy production and meteorological data	RF, SVM, CNN-LSTM	Denmark	RF showed the best performance, though its memory requirements during training may favor less resource-intensive methods.
[110]	2021	Short- & medium-term	2, 4, 6, 8, and 10 h ahead	Meteorological data, solar energy production data	SVM, GPR, BP, ELM, GWO-ELM, EGWO-ELM, IMF-EEMD-ELM	China	PV power forecasting under various weather conditions; the hybrid IMF-EEMD-ELM model achieved the highest accuracy.
[111]	2022	Short-term	Not specified	Historical solar irradiance data	LSTM-CNN	USA	The proposed algorithm outperforms other benchmark algorithms in 1-, 2-, 12-, and 24-step-ahead forecasts.
[112]	2022	Short-term	1 h ahead	Solar energy production data	LSTM, BiLSTM	Taiwan	BiLSTM shows better performance than traditional LSTM in PV generation forecasting.
[113]	2022	Short-term	1 h ahead	Solar energy data	PCC-LSTM	India	A hybrid model combining machine learning and statistics outperformed a machine learning-only model.
[114]	2022	Short-term	1 h ahead	Solar time series data	CNN-DeepESN	Australia	The model performs well in energy forecasting and generation, with MAE and RMSE values below 1%.
[115]	2023	Very short- & short-term	10 and 180 min ahead	Historical meteorological data, AC power output data	Optimized LSTM	Malaysia, Singapore	Univariate LSTM outperformed multivariate for short-term (10–50 min) forecasting by 2.09% RMSE. Multivariate performed better for longer horizons (180 min).
[48]	2023	Short-term	1 h ahead	Meteorological data	LSTM-GRU, BiLSTM-GRU, GRU-LSTM, 2GRU-BiLSTM, ADCMA-ResNet50-GRU-2LSTM	USA	The ADCMA-facilitated deep residual learning and gated recurrent network performed considerably better in 1 h ahead solar radiation prediction.
[116]	2023	Short-term	1 h ahead	Meteorological and PV data	TransNN-CNN	Vietnam	VMD decomposes input data in preprocessing. The proposed model outperforms five benchmark models with the lowest error values.
[117]	2023	Short-term	1 h ahead	PV data	SVR-LSTM	Portugal	Different base models perform best across different PV plants. Meta-learning can improve accuracy by up to 5% over the best base model per plant.
[118]	2023	Short-term	1 h ahead	Historical meteorological data	WOA-BiLSTM, WOA-BiLSTM-AM	Canada	The proposed WOA-BiLSTM-Attention model reduces RMSE by 56.09%, 80.10%, and 71.98% on cloudy, sunny, and rainy days compared to the baseline.
[119]	2023	Very short-term	30 min ahead	Historical PV output power	CEEMDAN-FIG-ILSTM-ARIMA	USA	First application of CEEMDAN-FIG-ILSTM-ARIMA to PV output interval forecasting; verified to have good performance.
[120]	2023	Very short- & short-term	5, 15, 30 min; 1, 6, and 24 h ahead	Meteorological data, GHI, DHR, RDT, RGT	VMD-ACNN	Australia	The proposed approach shows significant improvements and robustness in point and probabilistic forecasting tasks.
[121]	2023	Short-term	1 h ahead	Global irradiation dataset	NA-MEMD-LSTM1	India	Incorporating NA-MEMD with LSTM reduced average %RMSE compared to standalone LSTM and MEMD-based LSTM.
[122]	2023	Short- & medium-term	1, 10, 24, and 48 h ahead	Environmental parameters and PV plant generated energy	CNN-LSTM-BiLSTM	China	The hybrid approach achieves notable accuracy and resilience in energy production forecasting under varying weather conditions.
[123]	2024	Very short-term	30 min ahead	Historical PV energy data	XGBoost, PCC-LSTM	Not specified	PCC-LSTM achieved the best performance with the lowest RMSE and MAE values.
[72]	2024	Very short- & short-term	1 and 60 min ahead	Sky image data	ANN-LGBM	Brazil	GHI prediction using only whole-sky images; hybrid approach achieved ~30% higher average accuracy compared to traditional meteorological data.
[124]	2024	Very short- & short-term	30, 60, 90, and 120 min ahead	Historical active power, meteorological data, GHI	DCNN-BiLSTM	Australia	Using a pre-trained model addresses insufficient prediction accuracy caused by lack of operational data for PV stations.
[125]	2024	Short-term	1 h ahead	Historical active power, meteorological data	IVMD-IWOA-BiLSTM	Australia	The hybrid forecasting model reduces RMSE by 86.54%, 71.01%, and 72.71% on sunny, cloudy, and rainy days, respectively.
[126]	2024	Short-term	Not specified	PV generation data	CNN-BiGRU	China, Australia	The developed method exhibits exceptional forecasting performance and effectively improves PV generation prediction accuracy.
[127]	2024	Short-term	1 h ahead	PV generation data, meteorological data	WOA-VMD-SCINet	Australia	The proposed model outperforms eight other models, including LSTM, TCN, SCINet, and VMD-SCINet.
[128]	2024	Short- & medium-term	1, 2, 3, and 6 h ahead	Satellite data, NWP data	RFR-BiLSTM	Austria	Hyb + SAT + NWP is the optimal choice for intra-day hourly solar radiation forecasting.
[129]	2024	Short-term	1 h ahead	Mean temperature, sunshine duration, global radiation, PV generation	KNN-SVM	Not specified	The hybrid technique outperforms LSTM, improving accuracy by 98%.

**Table 5:** Comparative Analysis of IoT-Based Architectures for Photovoltaic Monitoring and Forecasting.

Ref.	Sensing Architecture	Communication Protocol	Edge Processing	Cloud Integration	AI/Forecasting Integration	Application Context
[76]	Voltage, current, irradiance, temperature sensors (ACS712, LM35, DHT) with Arduino/ESP8266	Wi-Fi	Basic preprocessing and data filtering	ThingSpeak/Cloud dashboard	Not integrated (monitoring only)	Residential PV monitoring
[77]	Multi-sensor distributed nodes	Wi-Fi/Gateway-based	Hierarchical preprocessing and semantic enrichment	Cloud analytics platform	Supports predictive analytics	Distributed energy systems
[78]	Electrical + meteorological sensors	GSM	Minimal edge computation	Remote server/cloud	Forecasting support for rural PV	Rural microgrids
[79]	Local sensor clusters	ZigBee	Low-power distributed sensing	Centralized monitoring server	No direct AI integration	Smart grid local networks
[80]	Solar performance metrics with IoT gateway	Wi-Fi/Cloud API	Real-time streaming	Cloud storage + visualization	Enables real-time forecasting	Remote solar plants

**Table 6:** Enabling technologies in hybrid models for short-term PV power forecasting.

Category	Algorithm	Hybrid Model	Critical Limitations	Refs
**Deep Learning Architectures (DL)**	LSTM, BiLSTM, GRU, 2GRU, CNN, CNN1D, ConvLSTM, DeepESN, ResNet50, SCINet, ILSTM, QRLSTM, TransNN	Captures complex nonlinear and temporal dependencies in PV data. Extracts spatial and spatio-temporal patterns from inputs like sky images. Handles noisy, highly variable signals common in solar generation.	High model complexity and risk of overfitting. High computational and data requirements. Performance can degrade under extreme weather variability without proper optimization.	[68,70,81,82,88,101,103,105,109,112]
**Evolutionary & Swarm Optimization**	GA, PSO, WOA, GWO, ALO, CSO, DHOA, GU-DHOA, DIFPSO, IWOA, EGWO	Optimizes hyperparameters and model architectures to enhance performance. Improves forecast robustness and accuracy under varying climate conditions. Reduces training instability and error in deep learning networks.	High computational cost due to population-based search. Results can be difficult to reproduce. Risk of over-optimization without improving real-world generalization.	[69,70,71,72,73,84,95,103,110,112]
**Signal Decomposition & Pre-processing**	CEEMDAN, EEMD, MEMD, NA-MEMD, VMD, IVMD, WT, WPD, EMD	Reduces noise and separates complex irradiance signals into simpler components. Facilitates learning for downstream deep learning models. Particularly improves performance for very short-term horizons (≤15 min).	Computationally expensive and can slow down the forecasting pipeline. May introduce artifacts if parameters are poorly tuned. Benefit is climate-dependent and may not always justify the added complexity.	[78,80,81,84,86,95,101,104,105,106,112]
**Hybrid Statistical–ML Frameworks**	ARIMA, ARMA-KF, VARX, Copulas (D-vine), PCC, FIG, ARIMA + DL	Captures residual temporal correlations not modeled by DL alone. Adds interpretability and statistical consistency to forecasts. Enhances the reliability of probabilistic forecasts.	Lower capacity to model highly complex nonlinearities. Dependent on statistical assumptions that may not hold in all conditions.	[77,85,86,88,98,104,108]
**Vision-Based & Remote Sensing Technologies**	Whole Sky Imagers (WSI), DCGAN, CNN (image-based), Satellite (SAT) data, NWP	Captures real-time cloud dynamics and movement for nowcasting. Crucial for ultra-short-term forecasting (1–15 min). Combining SAT and NWP data improves accuracy for 1–6 h horizons.	Very high computational resource requirements. Requires specialized camera infrastructure or satellite data access. Highly sensitive to local conditions (e.g., pollution, horizon view).	[5,59,60,83,92,93,109,113]

**Table 7:** Synthesis of deployment barriers and research directions for hybrid PV forecasting models.

Barrier Category	Specific Challenges	Proposed Solutions	Refs.
**Computational** **efficiency**	High training/inference cost; unsuitability for edge deployment	Lightweight hybrid architectures; model compression; cloud/edge co-design	[78,84,98,112]
**Data availability &** **quality**	Missing, noisy, or non-standardized data; regional biases	Transfer learning; synthetic data generation; federated learning frameworks	[43,59,77,109]
**Model interpretability & trust**	Black-box nature; lack of explainability in hybrid pipelines	Integration of XAI (SHAP, LIME); attention mechanisms; grey-box modelling	[81,89,119,128]
**Standardization &** **benchmarking**	Inconsistent metrics; lack of open datasets; reproducibility issues	Development of open benchmarks (e.g., PV-Net); adoption of probabilistic metrics	[30,55,104,121]
**Grid integration &** **interoperability**	Poor compatibility with SCADA/EMS; lack of real-time APIs	Modular design; standard protocols [IEEE 2030.5-2023]; containerized deployment	[20,21,126,131]
**Generalization across climates**	Overfitting to local data; poor performance in underrepresented regions	Cross-regional validation; physics-informed AI; adaptive training strategies	[5,62,85,111]

## Data Availability

The original contributions presented in this study are included in the article. Further inquiries can be directed to the corresponding author.

## Abbreviations

The following abbreviations are used in this manuscript:

AM	Attention mechanism
ANN	Artificial neural networks
AR	Auto regressive
ARIMA	Auto regressive integrated moving average
ARIMAX	Auto regressive integrated moving average
ARMA	Auto regressive moving average
BPNN	Back propagation neural network
CNN	Convolutional neural network
CONV	Convolution operation
CSO	Competitive swarm optimization
DGCA	Deep convolutional generative adversarial network
DIFPSO	Particle swarm optimization algorithm based on dynamic inertia factor
EGWO	Enhanced Gray wolf optimization
GA	Genetic algorithm optimization
GFM	Generalized fuzzy model
GHI	Global horizontal irradiation
GRUP	Gated recurrent unit pool
GWO	Grey wolf optimization
IGIVA	Improved grey ideal value approximation
IVMD	Improved variational mode decomposition
KF	Kalman filter
LGBM	Light gradient boosting machine
LSTM	Long short-term memory neural network
MED	Multivariate empirical mode decomposition
MLP	Multilayer perceptron network
NA	Noise-assisted
NN	Neural network
NWP	Numerical weather prediction
PI	Prediction interval
PSO	Particle swarm optimization
PV	Photovoltaic system
RBF	Radial basis function neural network
RNN	Recurrent neural network
SVM	Support vector machine
FS	Forecast skill
SVR	Support vector regression
IMF	Intrinsic mode function
WT	Wavelet transform
WOA	Whale optimization algorithm
WD	Wavelet decomposition
WPD	Wavelet packet decomposition
EMD	Empirical mode decomposition
SCA	Sine cosine algorithm
PCA	Principal component analysis
GBM	Gradient boosting machine
GRU	Gated recurrent unit
ML	Machine learning
MSE	Mean Squared Error
MAE	Mean absolute error
RMSE	Root mean square error
DHOA	Deer hunting optimization algorithm
R	Pearson correlation coefficient
s	Skill score
DL	Deep Learning
FE	Forecasting error
FOS	Flock-of-starlings optimization
ALO	Ant Lion optimization
ELM	Extreme learning machine
PDPP	Principles under a partial daily pattern prediction
IDBN	Improved deep belief network
VARX	Vector autoregressive model with Exogenous variables
QRLSTM	Quantile regression long short-term memory
VMD	Variational mode decomposition
PSFV	Prediction of Photovoltaic system
TS	Temporal series
